# Supplementary material for: Characterization and Molecular Dynamics Simulation of a Lipase Capable of Improving the Functional Characteristics of an Egg-Yolk-Contaminated Liquid Egg White
Source: Foods. 2023 Nov 11;12(22):4098. doi: 10.3390/foods12224098 (PMC10670289; doi:10.3390/foods12224098)
Supplement: Supplementary file 1 [file foods-12-04098-s001.zip › foods-2684187-supplementary.pdf]

Supplementary Material

Characterization and Molecular Dynamics Simulation of a Lipase Capable of Improving the Functional Characteristics of an Egg-Yolk-Contaminated Liquid Egg White

Linlin Xu [1], Fei Pan [2], Yingnan Li [3], Huiqian Liu [1] and Chengtao Wang [1,*]

[1] Beijing Advanced Innovation Center for Food Nutrition and Human Health, Beijing Engineering and Technology Research Center of Food Additives, Beijing Technology & Business University (BTBU), Beijing 100048, China; xll18515351711@163.com (L.X.); q2223599235@163.com (H.L.)

[2] State Key Laboratory of Resource Insects, Institute of Apicultural Research, Chinese Academy of Agricultural Sciences, Beijing 100093, China; yunitcon@yeah.net

[3] Ministry of Education Key Laboratory of Industrial Biotechnology, School of Biotechnology, Jiangnan University, Wuxi 214122, China; yingnan3400189@gmail.com

* Correspondence: wangchengtao@th.btbu.edu.cn; Tel.: +86-10-68984003

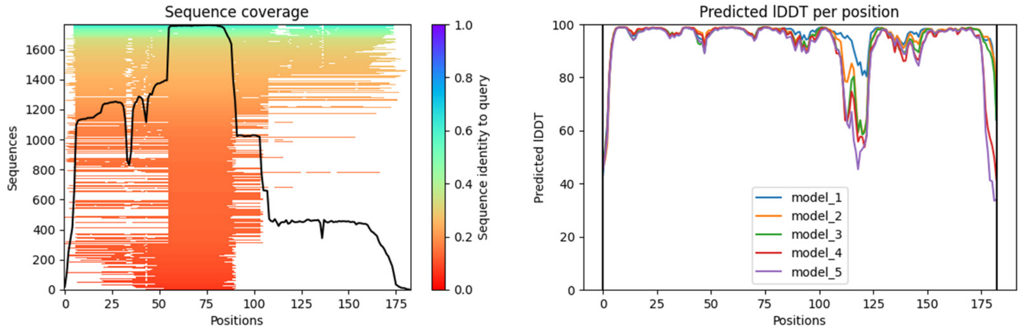

Figure S1. Evaluation of AlphaFold2 for predicting Lip-IM structure.
